# Untargeted metabolomics coupled with genomics in the study of sucrose and xylose metabolism in *Pectobacterium betavasculorum*

**DOI:** 10.3389/fmicb.2024.1323765

**Published:** 2024-05-15

**Authors:** Magdalena Smoktunowicz, Renata Wawrzyniak, Joanna Jonca, Małgorzata Waleron, Krzysztof Waleron

**Affiliations:** ^1^Department of Pharmaceutical Microbiology, Faculty of Pharmacy, Medical University of Gdańsk, Gdańsk, Poland; ^2^Department of Biopharmaceutics and Pharmacodynamics, Faculty of Pharmacy, Medical University of Gdańsk, Gdańsk, Poland; ^3^Laboratory of Plant Protection and Biotechnology, Intercollegiate Faculty of Biotechnology University of Gdańsk and Medical University of Gdańsk, University of Gdańsk, Gdańsk, Poland

**Keywords:** *Pectobacterium betavasculorum*, plant pathogen, metabolomics, GC-MS, sucrose, xylose

## Abstract

**Introduction:**

*Pectobacterium betavasculorum* is a member of the *Pectobacerium genus* that inhabits a variety of niches and is found in all climates. Bacteria from the *Pectobacterium* genus can cause soft rot disease on various plants due to the secretion of plant cell wall degrading enzymes (PCWDEs). The species *P. betavasculorum* is responsible for the vascular necrosis of sugar beet and soft rot of many vegetables. It also infects sunflowers and artichokes. The main sugar present in sugar beet is sucrose while xylose is one of the main sugars in artichoke and sunflower.

**Methods:**

In our work, we applied *metabolomic* studies coupled with genomics to investigate the metabolism of *P. betavasculorum* in the presence of xylose and sucrose as the only carbon source. The ability of the strains to use various sugars as the only carbon source were confirmed by the polypyridyl complex of Ru(II) method in 96-well plates.

**Results:**

Our studies provided information on the metabolic pathways active during the degradation of those substrates. It was observed that different metabolic pathways are upregulated in the presence of xylose in comparison to sucrose.

**Discussion:**

The presence of xylose enhances extracellular metabolism of sugars and glycerol as well as stimulates EPS and IPS synthesis. In contrast, in the presence of sucrose the intensive extracellular metabolism of amines and amino acids is promoted.

## 1 Introduction

The bacteria of the genus *Pectobacterium* inhabit a wide spectrum of ecological niches and have been isolated from soil, water, plants, and insects (Ma et al., 2007; Glasner et al., 2008). They can degrade plant cell walls through the secretion of plant cell wall degrading enzymes (PCWDEs) (Hugouvieux-Cotte-Pattat et al., 2014). *Pectobacterium* is considered as one of the top 10 bacterial pathogens that causes the harvest loss of potatoes and other vegetables both on the field and during transport or storage (Mansfield et al., 2012). However, representatives of this genus have also been found to cause disease symptoms of many other plants crops and ornamentals. The host range of inhabited and infected plants varies between *Pectobacterium* species (Ma et al., 2007; Charkowski, 2018).

**Figure 1:**
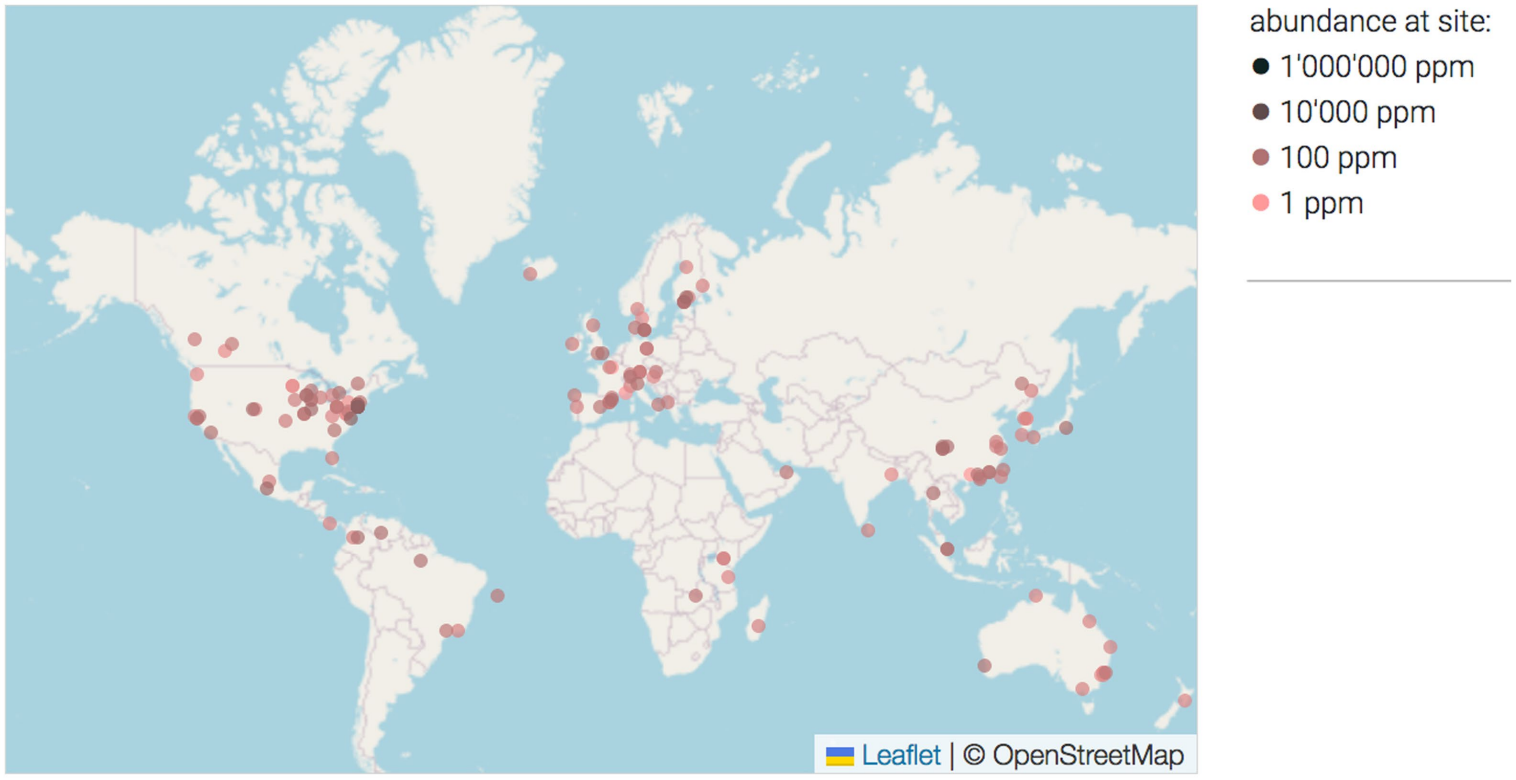
The map of locations where *P. betavasculorum* was found based on the 16S rRNA sequences from different environmental studies (Rodrigues et al., 2017). The intensity of the dot color reflect the aboundance on the site. **O =** 1 ppm, **O =** 100 ppm, **O** = 10,000 ppm.

Our attention caught the species *P. betavasculorum*, which is responsible for the vascular necrosis of sugar beet (Thomson et al., 1981). This group of bacteria was first described as a subspecies of *E. carotovora* subsp. *betavasculorum*, and then elevated to the species level *P. betavasculorum* (Gardan et al., 2003). The first records of this microorganism were from the USA, Mexico, La Reunion and Romania (Gardan et al., 2003). The species were recently isolated in Asia (Ozturk et al., 2019). *P. betavasculorum* is one of the most important causes of sugar beet root rot symptoms in Iran (Nedaienia and Fassihian, 2011), and South Korea (Jee et al., 2020). Based on the 16S rRNA sequences from different environmental studies, the map of locations recorded for this taxon was created (Figure 1) (Rodrigues et al., 2017). Its most favorable growth temperature is 28 ± 2°C. Disease symptoms in the form of black streaks and rotting develop within 2–10 days in leaves, stems, roots, fruits, and tubers, also in plants such as cucumber, bean, melon, tomato, squash, corn, potato, eggplant, carrots, turnips, garlic, and onions. Moreover, red beet and date palm fruit were presented as potential new hosts of *P. betavasculorum* (Saleh et al., 2008).

More detailed studies of the pathogenicity of *P. betavasculorum* revealed that this species causes rot and root vascular necrosis of sugar beet root and is casually isolated from sunflower, artichoke, and potato (Gardan et al., 2003). Due to its high sucrose content, sugar beet is a biennial plant used for sugar production. It reaches its highest sucrose content (15–20% of fresh weight) in the storage root at the end of the first, vegetative year of development (Getz, 2000). Sucrose is the primary sugar transported in the phloem of most plants (Stein and Granot, 2019). Xylose is the main component of xylan, a hemicellulose which is a major component of plant cell walls (Zhang et al., 2021). Xylan comprises about 30% of many economically important plants (birch for example). In the case of sunflower, it is 26 and 17.7% in artichoke (Fortunati et al., 2016; Pesce et al., 2020). Sunflower leaves and flowers contain glucose and xylose as the main sugars. Additionally, lesser amounts of galactose, mannose, and arabinose are also present (Fortunati et al., 2016). All those sugars are accessible to bacteria colonizing these plants, including *P. betavasculorum.*

Even though *P. betavasculorum* causes losses in sugar beet or sunflower, and thus in crops crucial for the production not only of food but also of raw materials for biofuels or biodegradable polymers, this species is not very well studied compared to other taxa. One reason may be that, so far, no rapid detection and identification methods have been developed. Therefore, the metabolism of *P. betavasculorum*, has not yet been well investigated by modern omics methods. The mere fact that *P. betavasculorum* is growing mostly on sugar beet may indicate a metabolism adapted for the colonization of this plant species (Borowska-Beszta et al., 2024).

Therefore, in our research, we conducted genomic and metabolomic studies in order to verify if *P. betavasculorum* strains adapted to inhabit and infect the plants containing high concentrations of sucrose or xylose, in their tissues. The observed metabolic changes, combined with biochemical and physiological tests, may enable pathway determination, regulatory inference and understanding of the adaptative abilities of *P. betavasculorum*.

## 2 Materials and methods

### 2.1 Materials

Strains of *P. betavasculorum* used in the study: B2 = IFB5269 = CFBP 2122^T^ = SCRI479 = NCPPB2795^T^ = 43762ATCC isolated from sugar beet; B5 = IFB5271 = CFBP1520 isolated from sunflower and B6 = IFB5272 = Sf142-2 isolated from artichoke. The strains were obtained from the Laboratory of Plant Protection and Biotechnology, Intercollegiate Faculty of Biotechnology of University of Gdansk and Medical University of Gdansk (IFB collection).

### 2.2 The ability to use sugars as the only organic carbon source

The ability of the strains to use sugars as the only carbon source was tested by the polypyridyl complex of Ru(II) method in 96-well plates (Jońca et al., 2021). For the experiment, 0.5% solutions of monosaccharides in the minimal medium M63 base were prepared (Miller, 1972). The following sugars were used: cellobiose, trehalose, sorbitol, sucrose, glucose and xylose. Aliquots 200 μL of the appropriate sugar solution, 2.5 μL of 5 mg/mL of ruthenium dye solution and 5 μL of a bacterial suspension of 0.5 McFarland (McF) optical density were added to each well. For each sugar solution, the sterility of the work was checked by adding only the nutrient solution and the ruthenium dye (negative control) to the well. The plates were incubated for 72 h at 28°C. Absorbance was read at 600 nm and in case of fluorescence samples were excited at 480 nm, and the fluorescence intensity was measured at 610 nm on the Infinite M200 Pro Tecan (Männedorf, Switzerland).

### 2.3 Testing the ability to form polysaccharides on a medium supplemented with various sugars

For the purposes of the experiment, a medium with the following composition was prepared: 2 g/L tryptone, 10 g/L sugar, 5 g/L sodium chloride, 1 g/L yeast extract, 0.300 g/L potassium hydrogen phosphate, 0.080 g/L bromothymol blue, 15 g/L agar. The final pH at 25°C was 7.1 ± 0.2. Sugars used in the experiment were glucose, mannitol, sucrose, galactose, xylose, maltose, ribose, rhamnose, isomaltulose. Bacteria grown overnight on Muller Hinton II (MHII) plates (Graso, Biotech, Poland) were suspended in the phosphate buffered saline (PBS) to the optical density of 0.5 McFarland (McF). The plates were inoculated by the spot method, 5 μL of the prepared bacterial suspension was placed on the plates. This allowed for a large number of bacteria to grow and produce metabolites at a single location. The plates were then incubated for 48 h. After that time, the plates were inverted and incubated again for 24 h to check for polysaccharide production.

### 2.4 Untargeted metabolomic analyses of biomass and media samples

For metabolomics analysis, bacteria were cultured on MS medium (Murashige and Skoog medium) without sugar content as a base. It is the most commonly used medium in laboratory experiments on plant tissue cultures and was selected to imitate the conditions of the *in vitro* plant growth. Carbon sources were added to the medium: 1% sucrose and 1% xylose, respectively. For each sugar, cultures were set in three biological replicates. Cultures were carried out for 72 h at 28°C and afterwards centrifuged (12,000 × g, 10 min). From the obtained supernatant, 1 mL of culture medium were taken in three technical replicates. Subsequently, the bacterial biomass samples were collected also in three technical replicates.

**Figure 2:**
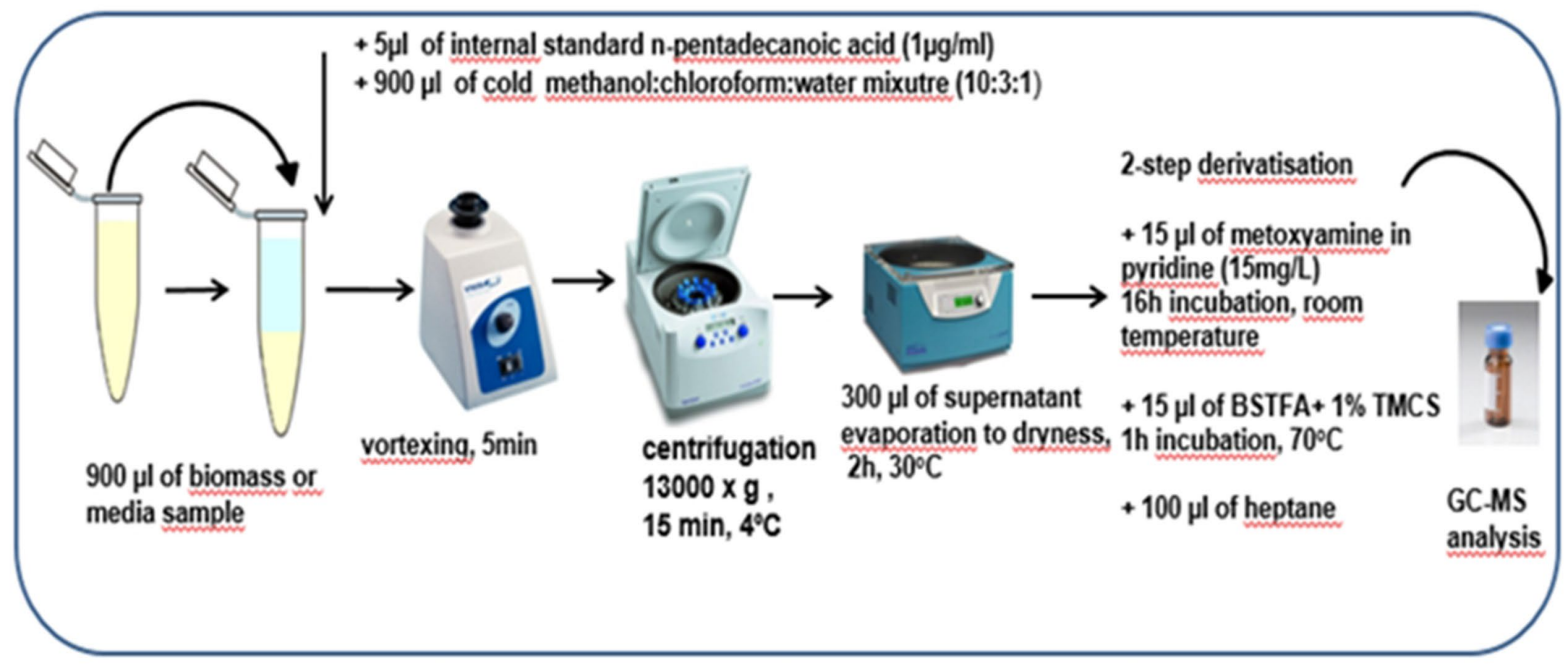
The detailed preparation procedure for biomass and media samples before analytical measurements with the use of GC-MS technique.

For the extraction of metabolites, 900 μL of methanol:chloroform: water mixture (10,3,1, v:v) were added to 900 μL of biomass from the bottom of the tube and to 900 μL of the nutrient medium after the bacteria cultivation. After centrifugation (13,000 × g, 15 min, 4°C), 300 μL of the obtained supernatants were concentrated by rotary vacuum and evaporated to the dryness. Afterwards, a two-step derivatization procedure was performed before analytical measurements with the use of gas chromatography–mass spectrometry (GC-MS) technique. The detailed procedure of sample preparation was displayed in Figure 2.

Quality Control (QC) samples, constituting a pool of an equal volume of all bacterial biomass or media samples, were prepared in the same manner as previously described (Figure 2). QC samples were analyzed throughout the sequence runs to ensure proper control of the system’s stability and analytical reproducibility.

The prepared bacterial biomass and media extracts were analyzed with the use of gas chromatography coupled with triple quadrupole mass spectrometry (GC-QqQ/MS) in two separate sequence runs. Chromatographic separations were performed with the use of Zebron ZB-5MS column (30 m × 0.25 mm, 0.25 μm). The GCMS-TQ8030 (Shimadzu, Japan) equipped with electron ionization source (EI) was used in the study. Both chromatographic and mass spectrometer parameters are described in detail in Table 1. The prepared bacterial biomass or media extracts were analyzed in a randomized order along with QC samples. Data acquisition was conducted using GC/MS Solution Software version 4.01 (Shimadzu, Japan).

**Table 1:** Chromatographic and mass spectrometer parameters of the GC-MS method.

Method parameter	The optimized value
Temperature gradient	60–320°C
Temperature gradient rate	8°C/min
Injection volume	1 μL
Injection temperature	250°C
Carrier gas	hel
Total flow rate of carrier gas	10 mL/min
Pressure of carrier gas	53.5 kPa
Interface temperature	300°C
Mass range (*m/z*)	50–600
Ion source voltage	70 eV
Ion source temperature	200°C

Peak detection and deconvolution of the acquired raw data were performed using the Automated Mass Spectrometry Deconvolution and Identification System (AMDIS) freeware^1^ (Stein, 1999; Du and Zeisel, 2013). The retention index (RI) for each detected compound was calculated by normalization of its retention time (RT) by the RT and RI of the closest eluting n-alkane, included in the standard mixture of even n-alkanes (C10-C40). This mixture was analyzed at the beginning of each sequence run. The deconvoluted data sets were multialigned with the use of MassProfiler Professional B.02.01 software (Agilent Technologies, Waldbronn, Germany). The obtained data matrices were normalized using intensity of internal standard. Identification of compounds detected with the GC-MS technique was based on RI, RT, and mass spectra present in the NIST 14 and in-house spectral libraries. Data matrices obtained after processing, include 9 samples (3 biological replicates in 3 technical replicates) for each growth condition. In the case of biomass extracts, data matrix contained 97 variables, namely detected metabolites. In the case of media extracts, dataset contained 114 variables, namely detected metabolites.

The Shapiro–Wilk test was first used to check the normality of data distribution and then *t*-test or U Mann–Whitney test (depending on data distribution) were applied. Additionally, to evaluate the homogeneity of variance between groups, Levene’s test was used. Subsequently, based on results of performed test the standard *t*-test in case of equal variances or Welch’s *t*-test in case of unequal variances were applied. Furthermore, the multivariate statistical analyses were performed, including Principal Component Analysis (PCA) and Orthogonal Partial Least Squares Discriminant analysis (OPLS-DA). PCA, as unsupervised method was performed to verify the quality of analytical measurements and general trends in the dataset. OPLS-DA is a supervised method which was applied to selected statistically significant variables which were able to discriminate the compared groups. All multivariate models were built on previously prepared datasets with the use of Pareto scaling and logarithmic transformation. The OPLS-DA models were k-fold cross-validated. The validation parameters included R^2^, Q^2^ and *p* CV-ANOVA. Statistical analyses, including both univariate and multivariate tests, were performed in Matlab (MATLAB 2016a, The MathWorks Inc., Natick, MA) and SIMCA P 16.0.1 (Sartorius stedim biotech, Umeå, Sweden) in order to evaluate metabolic changes in different growth conditions of *Pectobacterium betavasculorum*. The statistically significant metabolic changes observed in biomass and media extracts were selected based on both *p*-value < 0.05 after multiple testing with the use of FDR (False Discovery Rate) Benjamini-Hochberg method and Variable Importance in Projection (VIP) value > 1.0. Metaboanalyst 5.0^2^ a web-based platform were used for comprehensive interpretation of biochemical pathways in which identified metabolites are involved. MetaboAnalyst provides metabolic pathway evaluation by integration of pathway enrichment and pathway topology analyses.

### 2.5 Genomic analysis

Genomic and metabolomic analyses were performed with the use of Pathway Tools Software v27.0.^3^ As the reference, the publicly available genome of *P. betavasculorum* NCPPB2795^T^ strain isolated from sugar beet was used. All found metabolites were evaluated, including metabolites that were not statistically significant, with the use of Pathway Tools software.

## 3 Results

### 3.1 Testing the ability to use sugars as the only carbon source

Three strains were tested: B2 - isolated from sugar beet, B5 - isolated from sunflower; and B6 - isolated from artichoke. The bacterial growth and metabolic activity were tested on the following sugars: cellobiose, trehalose, sorbitol, sucrose, xylose, and glucose (Supplementary Figure S1). The highest *P. betavasculorum* growth and metabolic activity observed as a high absorbance (OD > 0.6), as well as high fluorescence (*F* > 50), were demonstrated in the medium with the addition of sorbitol (Supplementary Figure S1C) and sucrose (Figure 1D). Sugars that have been used as a carbon source for growth but have been used to a lesser extent for other metabolic processes are trehalose (Supplementary Figure S1B) and xylose (Supplementary Figure S1F). On the other hand, sugars used to support growth (OD = 0.3–0.4) and basic life processes (*F* < 16) are cellobiose (Supplementary Figure S1A) and glucose (Supplementary Figure S1E). Interestingly, the B6 strain isolated from artichoke showed higher metabolic activity on the sugars, while the B5 strain isolated from sunflower showed a higher growth rate and lesser fluorescence intensity than the B6 strain. The B2 strain, isolated from sugar beet, showed intermediate values of absorbance and fluorescence.

### 3.2 Testing the ability to form polysaccharides on a medium with various sugars

The ability of *P. betavasculorum* strains to produce exopolysaccharides in the presence of the following sugars was investigated: glucose, mannitol, sucrose, galactose, xylose, maltose, ribose, rhamnose and isomaltulose (Supplementary Table S1). The addition of bromothymol blue to the medium made it possible to observe the change in pH of the medium. The intensive production of mucus was observed on media supplied with two sugars: xylose and sucrose. The obtained results were used to design metabolomics studies.

### 3.3 Metabolic signatures of *Pectobacterium betavasculorum* in different growth conditions

Determination of both bacterial biomass and media metabolomic signatures was performed to evaluate the metabolite changes at intracellular and extracellular levels. After data processing, 97 and 114 metabolites were identified in bacterial biomass and media samples, respectively (Supplementary Tables S2, S3). When bacteria were cultivated in the presence of sucrose, 50 metabolites were common for metabolomic profiles of bacterial biomass and media extracts. These metabolites are derived mainly from carbohydrates and amino acid metabolic pathways. Exemplary metabolites that were unique for media extract include hypoxanthine, mannose, galactose, indole-3-pyruvic acid, adipic acid and docosenoic acid. On the other hand, metabolites that were detected only in bacterial biomass include, for instance, aconitic acid, maleic acid, phenylacetic acid and meso-erythritol. If xylose was the only source of organic carbon, 51 common metabolites associated mainly with carbohydrates and amino acids metabolism, were detected in both bacterial biomass samples and culture media extracts. The group of metabolites unique for culture media extracts contains, for instance, lactose, galactose, stearamide, indole-3-pyruvic acid, adipic acid and docosenoic acid. However, metabolites detected only in bacterial biomass samples include, for example, dihydroxybenzoic acid, aconitic acid, serine, maleic acid and 5-hydroxy-tryptophan.

**Figure 3:**
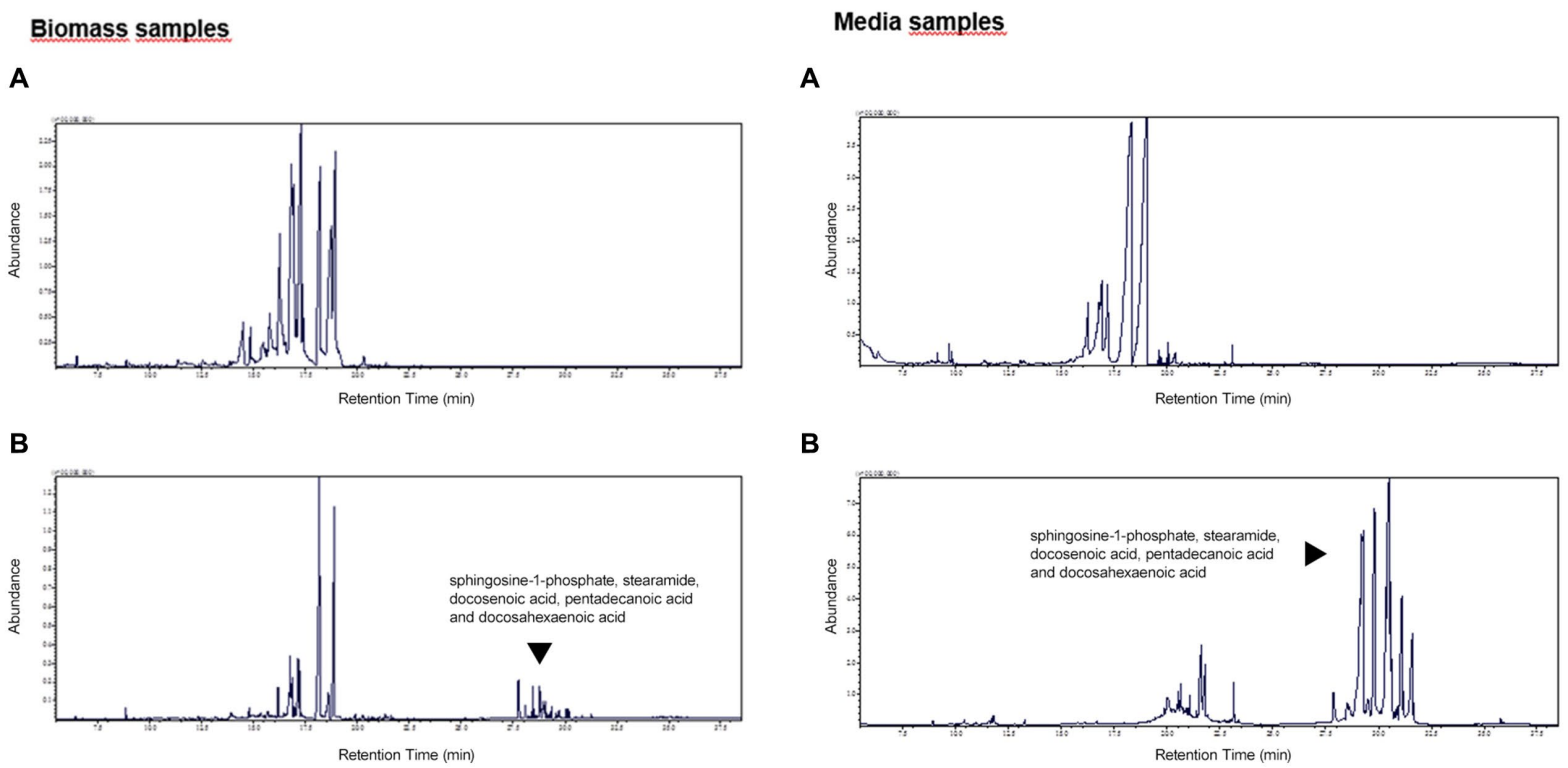
The exemplary metabolomic profiles for biomass and media extracts of *P. betavasculorum* in the presence of xylose **(A)** and sucrose **(B)**. The y-axis represents the abundance of detected compounds. The y-axis represents the retention time (RT) of detected compounds.

**Figure 4:**
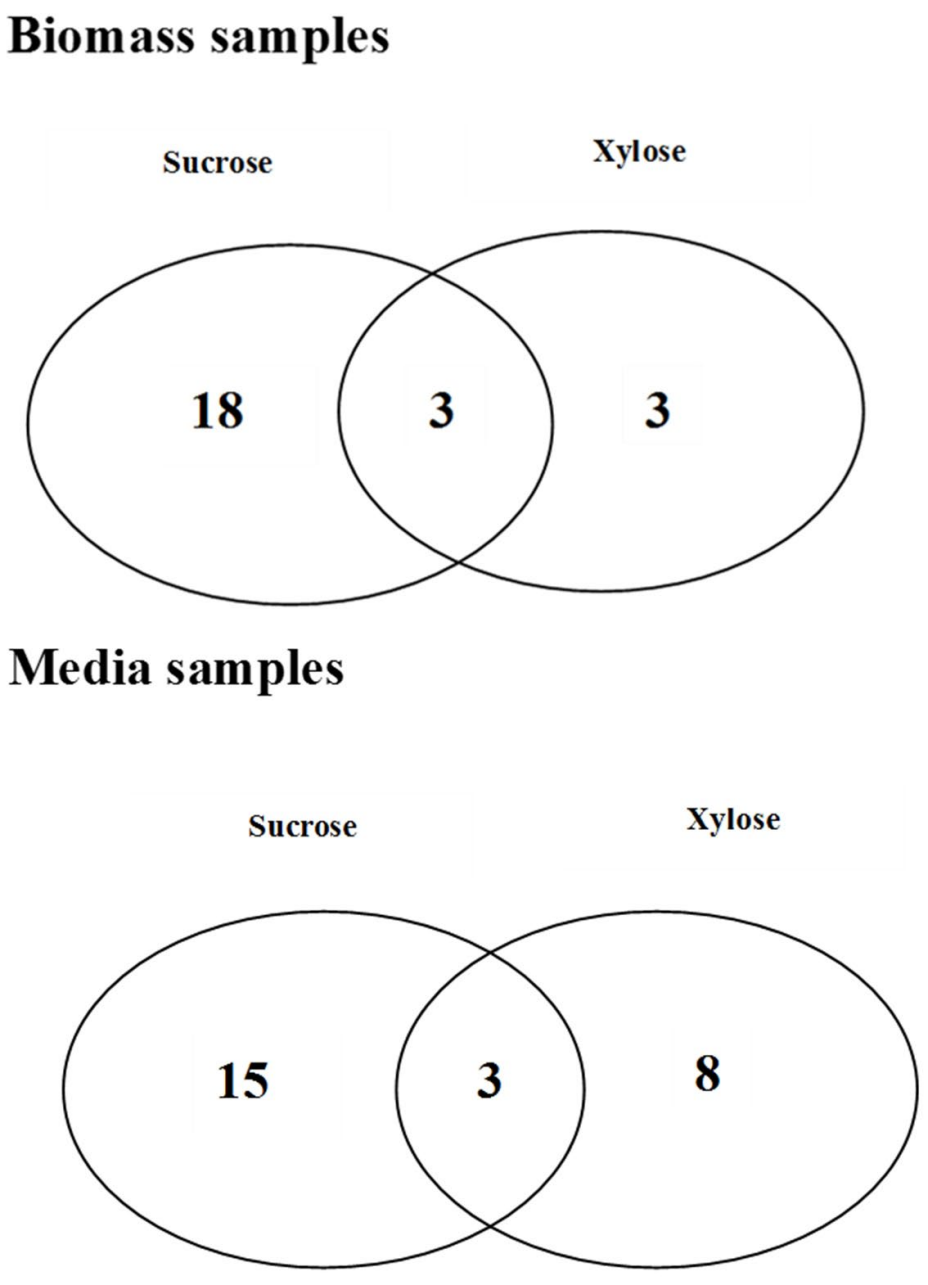
The Venn diagrams representing the number of common and unique metabolites which were found statistically significant in bacterial biomass samples and media extracts for different growth conditions.

The exemplary metabolomic profiles of biomass and media extracts in the presence of xylose or sucrose were presented in Figure 3. It can be noticed that in the presence of sucrose, the obtained metabolomic profiles for both bacterial biomass and media samples are enriched for more hydrophobic compounds, such as: sphingosine-1-phosphate, stearamide, docosenoic acid, pentadecanoic acid and docosahexaenoic acid, mainly related to fatty acid metabolism. As shown in Figures 3, 4, these compounds are more abundant in media samples which can be related to intensive extracellular metabolic reactions.

The metabolomic signatures of *P. betavasculorum* metabolism in different conditions of growth were compared and summarized in Tables 2, 3 as well as Figure 4. It should be underlined that more statistically significant metabolites were detected in the presence of sucrose than xylose, both in bacterial biomass (18 versus 3) and media extracts (15 versus 8). In the case of bacterial biomass, among selected statistically significant metabolites, three metabolites, namely, 5-hydroxy-L-tryptophan, arabinose and fructose were common for both growth conditions. However, in case of the media extracts, common metabolites for both growth conditions include creatinine, galactofuranose and glucopyranose. The significant metabolites that were unique if xylose constituted the only source of organic carbon, include lyxose, sucrose and xylitol in the bacterial biomass samples as well as norvaline, glycerol, arabinopyranose, threitol, mannopyranoside, arabinose, propanediamine and nicotinic acid mononucleotide in culture media extracts. The results of PCA and validated OPLS-DA models were presented in Supplementary material section 2.

**Table 2:** Statistically significant metabolites detected in bacterial biomass samples of *Pectobacterium betavasculorum* cultivated in the presence of xylose or sucrose as a source of organic carbon.

Metabolite	RT	Identification	VIP	*p* value
Butane	9.9	103,73,45	1.47	0.000333
8,10-Dioxaheptadecane	12.3	129,97,57	1.49	0.000333
2-Methylbutyrate	12.6	159,75,73	1.48	0.000333
2-Hydroxyisocaproic acid	14.8	189,117,75	1.55	0.000333
Glycerol	15.2	293,205,147	1.43	0.000333
Galactofuranose	16.6	319, 217,73	1.61	0.000333
Erythro-Pentonic acid	17.2	306,147,73	1.6	0.000547
d-Ribose	17.3	305,191,217	1.28	0.021198
Lyxopyranoside	17.9	305,204,133	1.42	0.000333
Threitol	18.1	307,205,103	1.2	0.021198
Ribitol	18.4	319,307,103	1.65	0.000547
2-Deoxy ribose	18.6	218,129,73	1.5	0.000333
Serine	18.8	218,204,73	1.55	0.000547
5-Hydroxy-L-tryptophan	18.8	220,204,146	1.47	0.000333
Arabinose	18.9	333, 217,191	1.5	0.000333
Mannose, 6-deoxy	18.9	393,204,73	1.39	0.000333
Arabinopyranose	19.1	333,217,204	1.38	0.000333
Gulose	19.8	435,204,73	1.48	0.000333
Glucopyranoside	19.8	409,187,101	1.35	0.000333
Fructose	20.1	437,204,73	1.36	0.000333
Mannopyranose	20.2	377, 204,73	1.08	0.006096
Lyxose	20.7	333,204,73	1.33	0.014908
Sucrose	28.6	361,217,73	1.26	0.000333
Xylitol	29.0	307,103,73	1.54	0.000333

Metabolites detected only in presence of sucrose are marked in green. Metabolites detected only in presence of xylose are marked in blue. Metabolites common for both growth conditions are marked in orange. RT, retention time; VIP, variable importance in projection.

**Table 3:** Statistically significant metabolites detected in media extracts of *Pectobacterium betavasculorum* cells cultivated in the presence of xylose or sucrose as a source of organic carbon.

Metabolite	RT	Identification	VIP	*p* value
4-Imidazoleacrylic acid (Trans-urocanic)	9.9	309,235,73	1.21	0.011671
Norvaline	10.7	251,108,91	1.14	0.020229
Glycerol	15.1	293,205,147	1.41	1.7 × 10–4
Creatinine	15.3	329,115,73	1.09	0.00388
Arabinopyranose	18.4	305,207,204,73	1.48	1.7 × 10–4
Threitol	18.4	307,205,103	1.38	1.7 × 10–4
Mannopyranoside	18.6	377,204,73	1.5	1.7 × 10–4
Arabinofuranose	18.9	393,217,73	1.29	1.7 × 10–4
2-Deoxy ribose	18.9	218,129,73	1.39	0.006291
Galactofuranose	18.9	319,217,73	1.03	0.009699
Arabinose	19.2	333, 217,191	1.32	0.006291
Fructose	19.4	437,204,73	1.51	1.7 × 10–4
Glucopyranoside	19.8	409,187,101	1.48	1.7 × 10–4
Sorbose	20.3	437,204,73	1.5	1.7 × 10–4
Galactose	20.6	320,204,191,73	1.49	1.7 × 10–4
Galactinol	20.8	421,307,217	1.15	0.000174
Hypoxanthine	20.8	280,265,73	1.18	0.006291
Mannose	20.8	204,191,73	1.17	0.020229
Talose	20.8	217,204,191,73	1.51	1.7 × 10–4
Glucopyranose	21.01	204,191,147	1.04	0.000339
Propanediamine	21.1	196,154,72	1.06	0.038965
Galactofuranoside	22.4	217,205,101	1.15	0.006291
Maltose	27.8	361,204,191	1.39	1.7 × 10–4
Sucrose	28.1	361,217,73	1.45	1.7 × 10–4
Lactose	29.4	361,204,73	1.43	1.7 × 10–4
Nicotinic acid mononucleotide	31.02	235,147,73	1.29	0.006476

Metabolites detected only in presence of sucrose are marked in green. Metabolites detected only in presence of xylose are marked in blue. Metabolites common for both growth conditions are marked in orange. RT, retention time; VIP, variable importance in projection.

**Figure 5:**
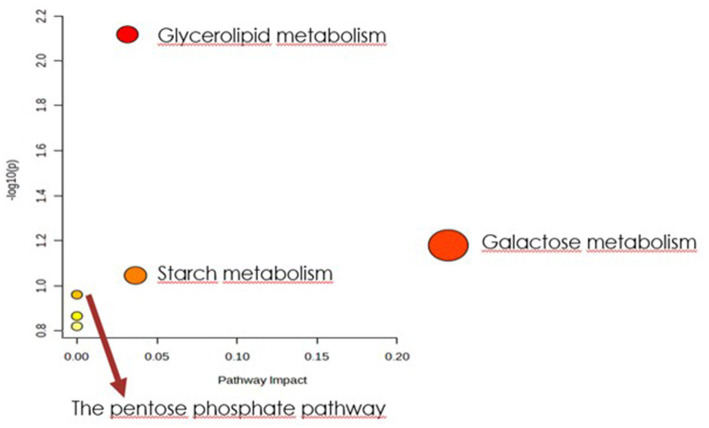
Biochemical evaluation of metabolic pathways in bacterial biomass samples. The x-axis represents the pathway impact value computed with the use of pathway topological analysis. The y-axis is the-log of the *p*-value obtained from pathway enrichment analysis. The pathways that were most significantly changed are characterized by both a high-log(p) value and high impact value (top right region).

**Figure 6:**
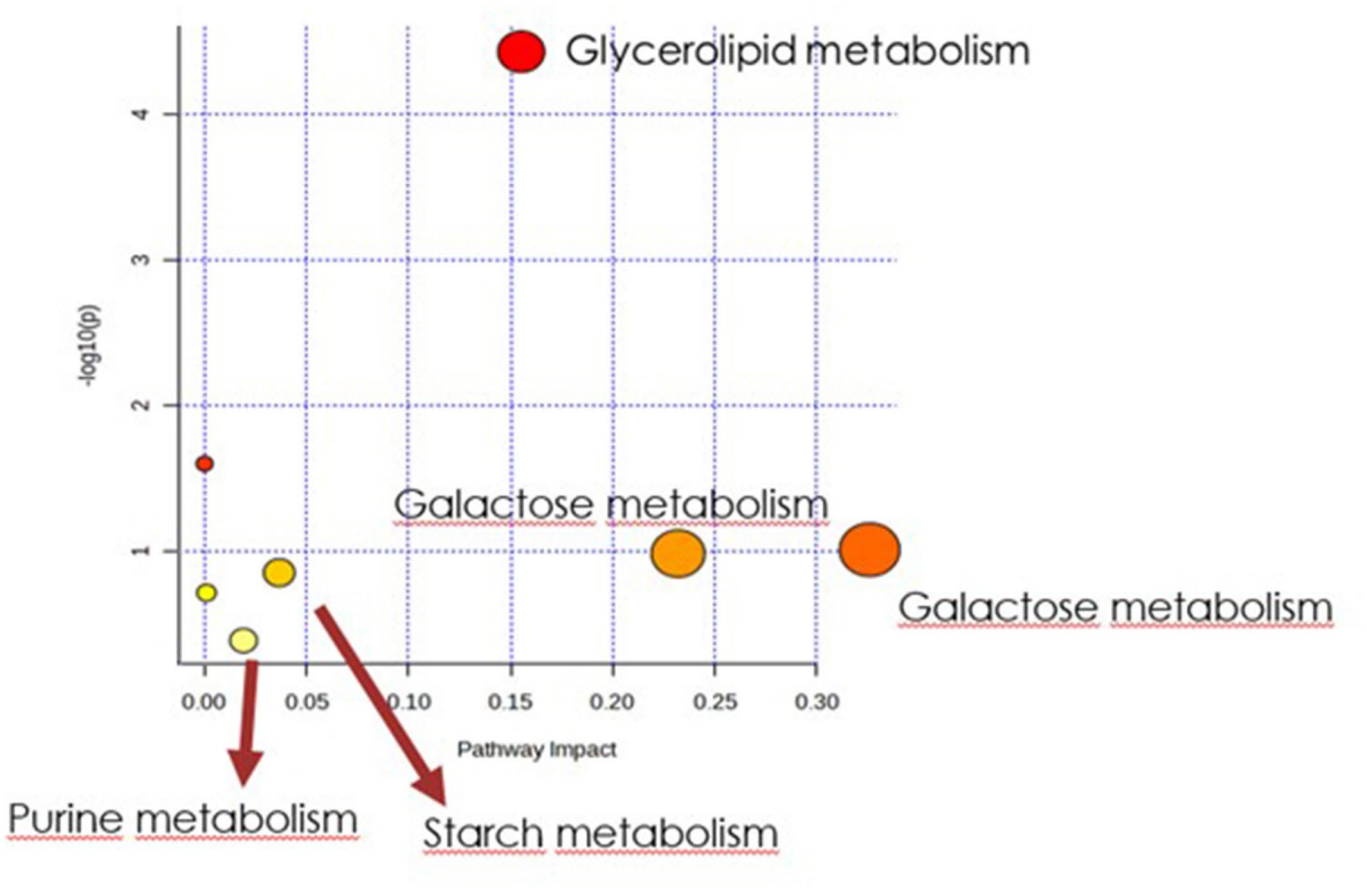
Biochemical evaluation of metabolic pathways in culture media extracts. The x-axis represents the pathway impact value computed with the use of pathway topological analysis. The y-axis is the log of the *p*-value obtained from pathway enrichment analysis. The pathways that were most significantly changed are characterized by both a high-log(p) value and high impact value (top right region).

In order to identify the network of the observed metabolite changes, the pathway analysis in Metaboanalyst 5.0 (see footnote 2) was performed (Figures 5, 6). Observed metabolite changes in the presence of xylose and sucrose in bacterial biomass samples were related mainly to glycerolipid, galactose, starch metabolism and pentose phosphate pathway reactions. The changes were similar in the media extracts with the exception of additional differences in purine metabolism (Figure 6).

**Figure 7:**
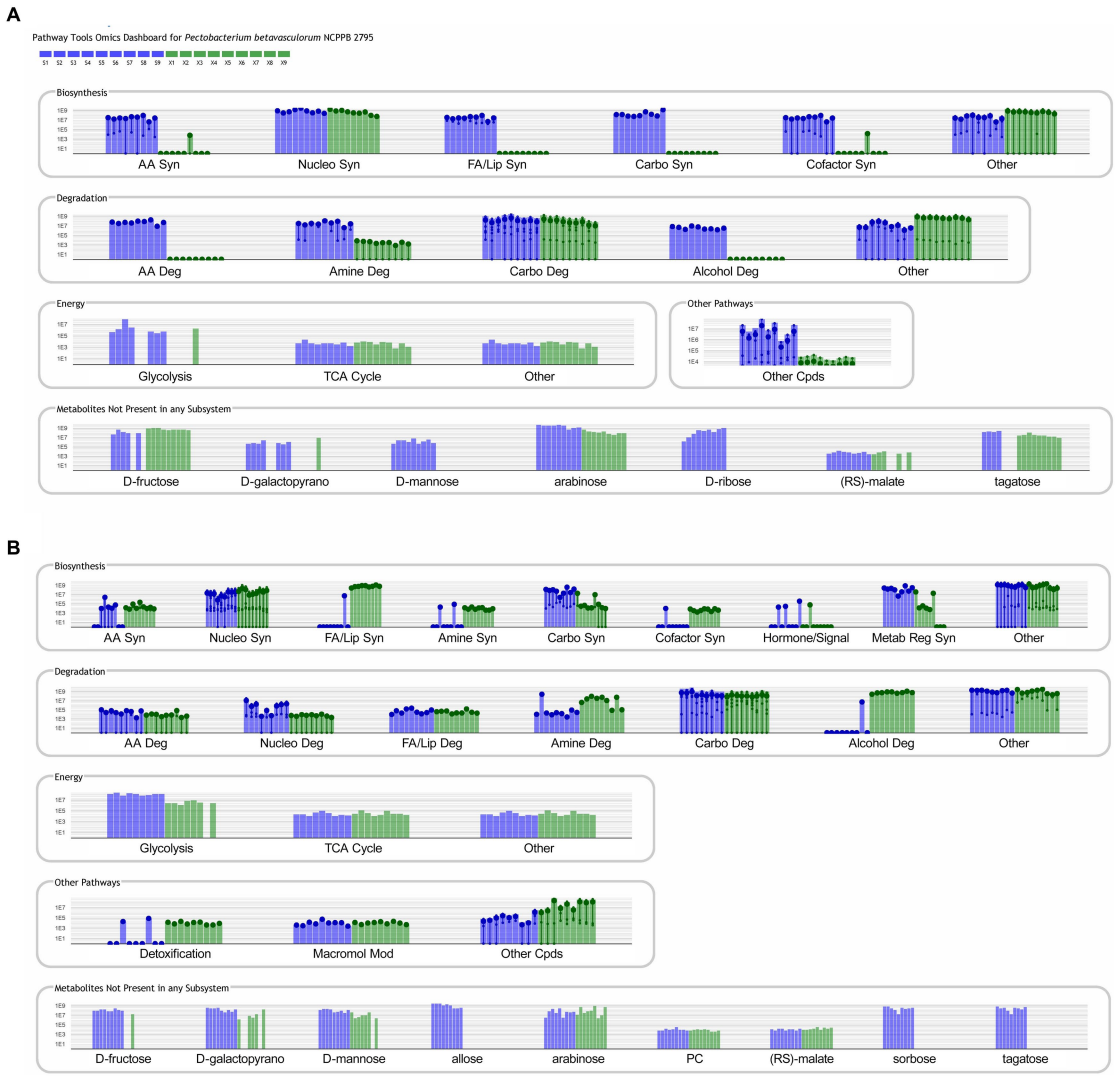
Metabolic pathways identified in PathwayTools *P. betavasculorum* NCPPB 2795 genome database based on the biomass **(A)** and medium **(B)** metabolomics data. Blue color indicates sucrose containing samples whereas green pertains to the samples supplemented with xylose. Each experiment was repeated three times and conducted in triplicate.

Obtained results were additionally analyzed using the Pathway Tools Software v27.0.^4^ For culture media extracts and biomass data, 33 and 25 compounds were identified in the custom database created from *P. betavasculorum* NCPPB2795^T^ genome, respectively (Supplementary Tables S4, S5). The analysis revealed that in the biomass samples, metabolic processes involved in alcohol degradation and lipid synthesis were only active in sucrose-containing medium (Figure 7A). The opposite could be observed in case of bacterial medium (Figure 7B). Other processes that showed a greater activity in sucrose-containing biomass samples than xylose-containing biomass samples were processes of amino acid synthesis and degradation, and cofactor synthesis (Figure 7A). The metabolomic datasets were also analyzed for Pathway Perturbation Score (PPS) and Differential Pathway Perturbation Score (DPPS) which attempt to measure the overall extend of pathways down or upregulation. The 20 pathways of the highest DPPS detected in biomass extracts containing sucrose were listed in Supplementary Table S6 and visualized in Supplementary Figure S2. They included pathways of sucrose and glycogen degradation. In biomass extracts containing xylose, those pathways were also upregulated (Supplementary Table S7; Supplementary Figure S3). Media extracts of sucrose-containing samples indicated the activity of enzymes involved in purine metabolism and upregulation of nucleotide degradation pathways as well as pathways of cardiolipin and phospholipids biosynthesis (Supplementary Table S8; Supplementary Figure S4). In contrast, media extracts containing xylose had increased activity of enzymes involved in the metabolism of sugars such as sucrose, trehalose, mannose, D-xylose and lactose (Supplementary Table S9; Supplementary Figure S5). Glycogen degradation pathway was also upregulated.

## 4 Discussion

*P. betavasculorum* is unique among *Pectobacterium* genus in its ability to colonize plants which contain high sucrose (sugar beet) and xylose (sunflower and artichoke) content (Borowska-Beszta et al., 2024). In our work, we conducted metabolomic and genomic studies to shed some light on its adaptation potential and metabolic pathways which become upregulated in the presence of those sugars. Our investigation showed that *P. betavasculorum* strains were able to utilize both, sucrose and xylose as the only carbon source. It is indicative of their ability to colonize hosts with a high content of those sugars.

*P. betavasculorum* was able to grow on xylose and sucrose as the only organic carbon source (Supplementary Figures S1D,F). Genomic analysis confirmed the presence of sucrose and xylose degradation pathways in its genome (Supplementary Figure S6) as well as the pathway of glycogen degradation (Supplementary Figure S7). The results of growth assays agreed with the metabolomic, coupled with genomic, analysis of pathways upregulated during bacterial growth. In general, *P. betavasculorum* strains exhibited higher metabolic activity in the presence of sucrose than xylose. Those results were not surprising as sucrose provides a more readily available source of energy for growth as it is a dimer of glucose and fructose.

Sucrose can be transported into the cell and hydrolysed, or it can be hydrolysed extracellularly and converted into polymers (Gering and Brückner, 1996). For example, *Erwinia amylovora* can utilize sucrose via secreted levansucrase (Bogs and Geider, 2000). The main classes of enzymes involved in the breakdown of sucrose are hydrolases and phosphorylases whereas glycosyl-nucleotide glycosyltransferases synthesize carbohydrate polymers (Reid and Abratt, 2005). These polymers act as pathogenicity factors or can serve as an alternative energy source when other nutrients become depleted (Gering and Brückner, 1996).

Extracellular sucrose can enter to the periplasm of Gram-negative bacteria via channels in the outer membrane created by porins, then is transported through the inner (cell) membrane. The sucrose-related pathway includes the membrane-bound phosphoenolpyruvate:sugar phosphotransferase systems (PTS), which causes sugar transport as well as phosphorylation (Costa-Riu et al., 2003). The hydrolysis of the phosphorylated disaccharide in the cytosol is performed by enzymes with sugar specificity, such as sucrose hydrolase. The product β-D-glucose-6-phosphate can enter glycolysis, and D-fructose-6-phosphate is an intermediate in glycolysis (Meadow et al., 1990).

The gene of the PTS transporter (KP22_RS13800), as well as sucrose-6-phosphate hydrolase (KP22_RS13795), are both present in the *P. betavasculorum* genome (Supplementary Figure S5). What is more, metabolomic analysis of bacterial biomass confirmed that this pathway was upregulated (Supplementary Figure S2). Another upregulated pathway involved in sucrose catabolism was a hydrolysis of sucrose-by-sucrose invertase [β-D-fructofuranoside fructohydrolase (EC 3.2.1.26)], the product of the KP22_RS13795 gene of *P. betavasculorum*. Unsurprisingly, the glycolysis pathway was also upregulated.

The enzymes responsible for intracellular polysaccharide degradation or synthesis are encoded by the operon *glg.* This includes *glg*A (glycogen synthase), *glg*B (branching enzyme), *glg*C and D (two subunits of ADP-Glc pyrophosphorylase, ADP-Glc-PP) and *glg*P (or *phs*G, glycogen phosphorylase). The crucial regulating point of intracellular polysaccharide (IPS) biosynthesis is the generation of ADP-glucose, catalyzed by ADP-Glc-PP. This reaction is allosterically regulated by the intermediates, activators fructose-6-phosphate (F6P) or fructose-1,6-bisphosphate (F-1, 6-bP) and inhibitors AMP, ADP or Pi. This corresponds to the conditions of high and low energy supply, respectively. Studies have shown that transcript levels of the *glg* operon are upregulated under glucose-limiting conditions and downregulated with excess of glucose. However, the presence of sucrose results in decreased *glg* operon expression compared to glucose. Sucrose is usually converted into soluble and insoluble exopolysaccharides (EPS) by glucosyltransferases, forming glucose homopolymers called glucans. Glucans promote bacterial attachment and biofilm accumulation, which undoubtedly enhances the virulence of the species (Staat and Schachtele, 1974; Burne et al., 1996; Khalikova et al., 2005; Klahan et al., 2018). It has been shown that the activity of these exoenzymes, as well as the internalization of sucrose by PTS, have a profound effect on the regulation of bacterial genes, both through the release of monosaccharides, as glucose or fructose, and PTS-dependent regulation of gene expression (Verhaeghe et al., 2021).

*P. betavasculorum* contains the *glg* operon genes in its genome and a complete pathway of glycogen synthesis and degradation (Supplementary Figure S7). Metabolomic analysis revealed that this pathway was upregulated in the presence of sucrose in bacterial biomass (Supplementary Figure S2). It suggests the production of IPS. The pathway was not found upregulated in the bacterial media samples (Supplementary Figure S4). However, our studies show that *P. betavasculorum* produces EPS in a sucrose-supplemented medium. It may suggest a different EPS composition than IPS.

Several other pathways were also found upregulated. N-acetylglucosamine is a component of bacterial cell wall. However, it can also play a role in cell signaling in bacterial pathogenesis (Ansari et al., 2022). What is more, poly-β-1,6-N-acetyl-d-glucosamine (PGA) is a crucial component of *Pectobacterium* biofilm (Pérez-Mendoza et al., 2011). Cardiolipin is a component of bacterial membranes. It is synthesized in response to osmotic stress to which bacteria are undoubtedly subjected during infection of sugar beet root (Romantsov et al., 2009). Putrescine and agmatine provide protection against acid stress (del Rio et al., 2016). Upregulation of those pathways may play a role in *P. betavasculorum* adaptation to colonization of sugar beet. It is worth noting that neither agmatine nor putrescine pathways were found upregulated in xylose-supplemented media samples.

Many bacteria species use an active transport system for the uptake of xylose into the cell (Oh and Kim, 1998). Xylose is initially converted to xylulose. In the case of bacteria, xylose to xylulose isomerisation process is performed by xylose isomerase (Zhao et al., 2020). Xylose is utilized through three alternative metabolic pathways: the isomerase pathway; the oxidoreductase pathway; and the oxidative pathway, also called as the non-phosphorylative pathway. Among the bacteria of the order *Enterobacteriales*, the isomerase pathway is the main pathway. The isomerase pathway converts xylose into xylulose, then it is phosphorylated to xylulose phosphate an intermediate of the pentose phosphate pathway (Domingues et al., 2021). Genes coding for xylose isomerase and phosphatase enzymes are induced by xylose and repressed by glucose as well as the other usable substrates, according to the to use xylose as the carbon source (Vivek et al., 2022). This pathway is characteristic for prokaryotes (Lawlis et al., 1984; Lokman et al., 1991; Rygus et al., 1991; Stephens et al., 2007). The oxidoreductase pathway is present mainly in eukaryotic organisms where the xylose is converted to xylitol, which is subsequently dehydrogenated and phosphorylated to xylulose phosphate, entering the pentose phosphate pathway (Wong et al., 1991; Desai and Rao, 2010).

*P. betavasculorum* possesses genes of xylose isomerase *xyl*A as well as xylulokinase *xyl*B (Supplementary Figure S6), which together convert D-xylose to D-xylulose-5-phosphate. It was consistent with the metabolomic studies results as this pathway was found upregulated in bacteria grown in xylose supplemented medium (Supplementary Figure S3). Moreover, a putative xylose reductase gene (WP_039325299) is present in *P. betavasculorum* genome, suggesting that this bacterium can produce xylitol from xylose. Xylitol was indeed detected in bacteria grown in xylose supplemented medium samples. The xylose reductase pathway is more thermodynamically advantageous than the xylose isomerase pathway and thus allows faster assimilation of xylose (Mouro et al., 2020). It may be the adaptation of *P. betavasculorum* to infecting plants with high lignocellulose content.

Sucrose degradation pathways as well as pathways involved in glycogen metabolism were found to be upregulated during growth in xylose-supplemented medium. It suggests intensive production of IPS and EPS. Those findings agree with phenotypic tests which confirmed that in the presence of xylose, *P. betavasculorum* strains produced significant amounts of EPS. Produced EPS may differ in composition to the EPS produced in sucrose supplemented medium. Such differences in EPS composition might be due to a host specific adaptation. However, to support this hypothesis, further studies on the composition of EPS derived from different sugars are necessary.

In the presence of xylose, *P. betavasculorum* exhibited enhanced extracellular metabolism of sugars and glycerol. In contrast, the presence of sucrose promoted intensive extracellular metabolism of amines and amino acids. The latter change in the metabolism the *P. betavasculorum* might maintain proper intracellular carbon and nitrogen levels, that is crucial to maximize nutrient utilization and cell growth. Among systems regulating carbon and nitrogen pools are the regulatory phosphotransferase systems that serve as a sensor responsible for metabolism tuning depending on the nutrients availability. It has been shown that PTS is required for EPS production and controls central carbon metabolism via the tricarboxylic acid (TCA) cycle (Sánchez-Cañizares et al., 2020).

To conclude, untargeted metabolomic studies combined with physiological and genomic analyses provided valuable information about metabolic pathways upregulated during the growth in the presence of xylose and sucrose, two main sugars found in *P. betavasculorum* native hosts. Our studies revealed that bacteria from the *P. betavasculorium* species may successfully utilize sucrose and xulose for growth and metabolism, and the formation of both, IPS and EPS.

## Data availability statement

The original contributions presented in the study are included in the article/Supplementary material, further inquiries can be directed to the corresponding authors.

## Author contributions

MS: Conceptualization, Data curation, Methodology, Writing – original draft. RW: Conceptualization, Data curation, Methodology, Visualization, Writing – original draft, Writing – review & editing. JJ: Methodology, Data curation, Visualization, Writing – review & editing. MW: Conceptualization, Data curation, Funding acquisition, Supervision, Project administration, Writing – review & editing. KW: Conceptualization, Data curation, Funding acquisition, Supervision, Project administration, Writing – review & editing.

## Footnotes

^1^ http://www.amdis.net

^2^ http://www.metaboanalyst.ca

^3^http://bioinformatics.ai.sri.com/ptools/, accessed on 12 October 2023.

^4^http://bioinformatics.ai.sri.com/ptools/, accessed on 14 August 2023.
