# Supplementary material for: Untargeted metabolomics coupled with genomics in the study of sucrose and xylose metabolism in Pectobacterium betavasculorum
Source: Front Microbiol. 2024 May 15;15:1323765. doi: 10.3389/fmicb.2024.1323765 (PMC11133636; doi:10.3389/fmicb.2024.1323765)
Supplement: Supplementary file 2 [file Table_2.DOCX]

Supplementary Material

# Supplementary Figures and Tables

## Supplementary Figures


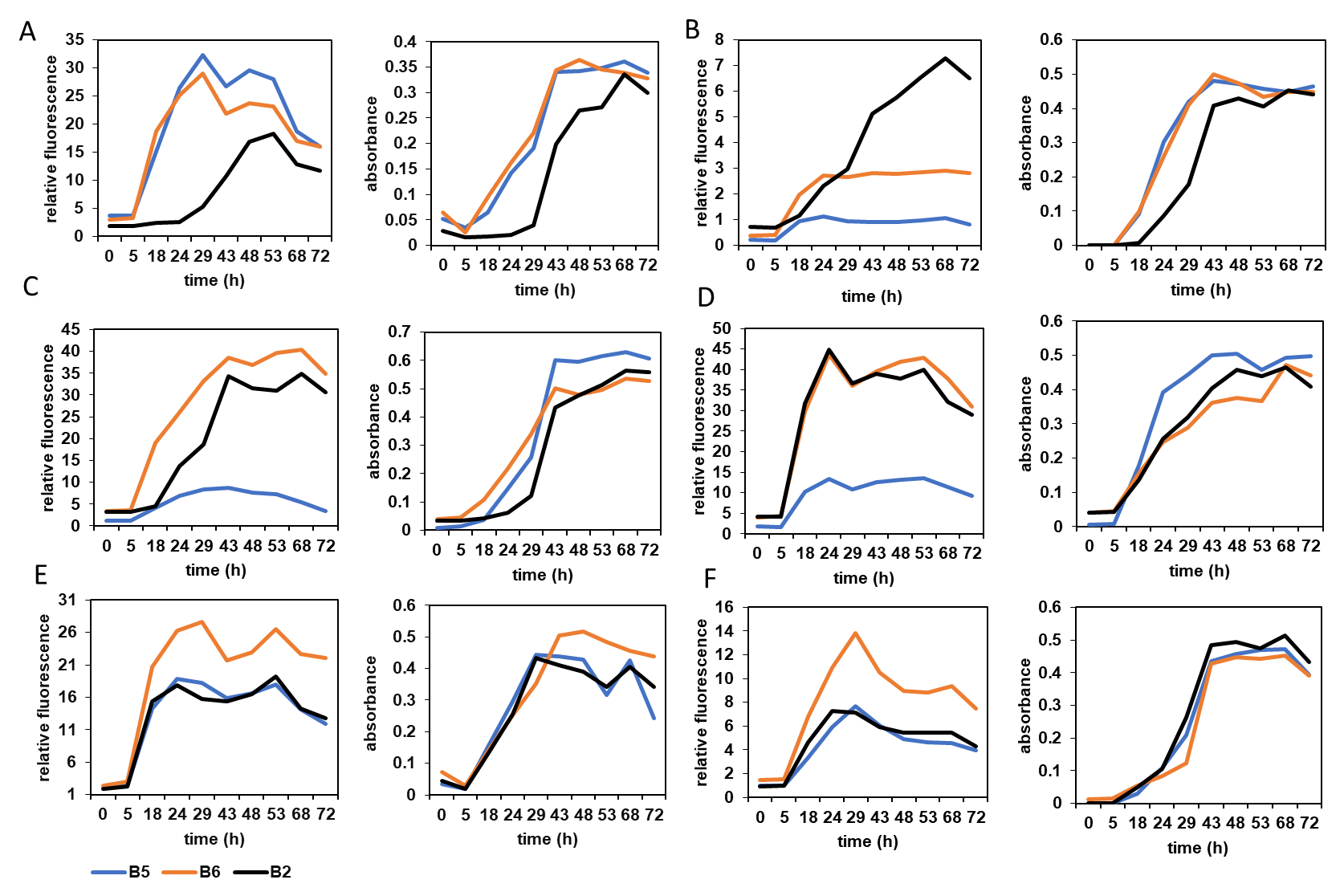


**Supplementary Figure 1.** The metabolic activity and growth of *P. betavasculorum* strains in media supplemented with cellobiose (A), trehalose (B), sorbitol (C), sucrose (D), glucose (E) and xylose (F).


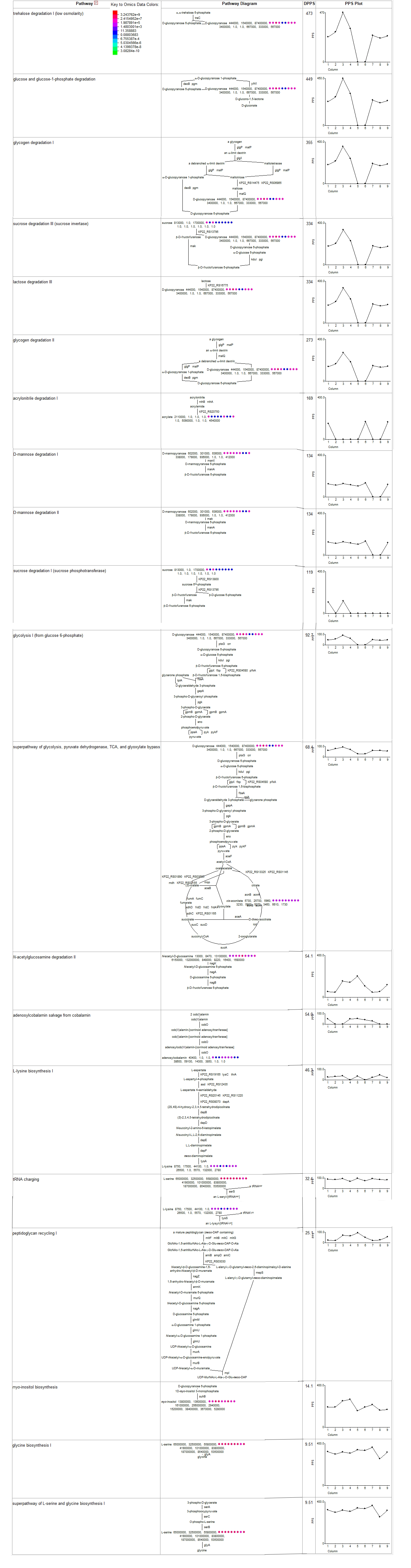


**Supplementary Figure 2.** The pathways detected in *P. betavasculorum* NCPPB 2795 genome database after analysis against compounds present in bacteria grown in sucrose supplemented medium. The analysis was performed in PathwayTools software.


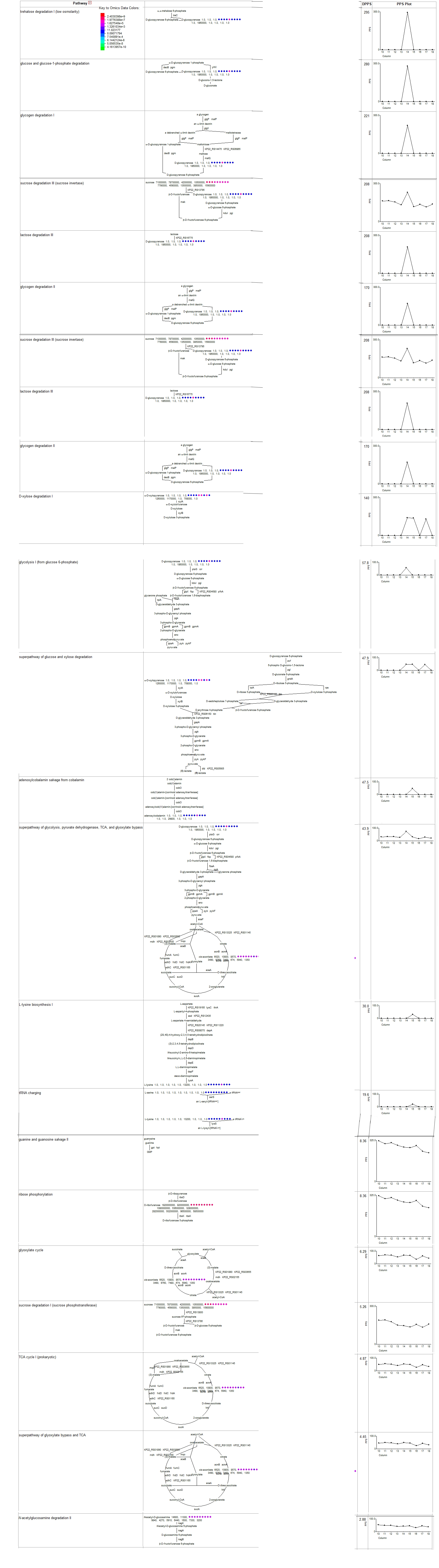


**Supplementary Figure 3.** The pathways detected in *P. betavasculorum* NCPPB 2795 genome database after analysis against compounds present in bacteria grown in xylose supplemented medium. The analysis was performed in the PathwayTools software.


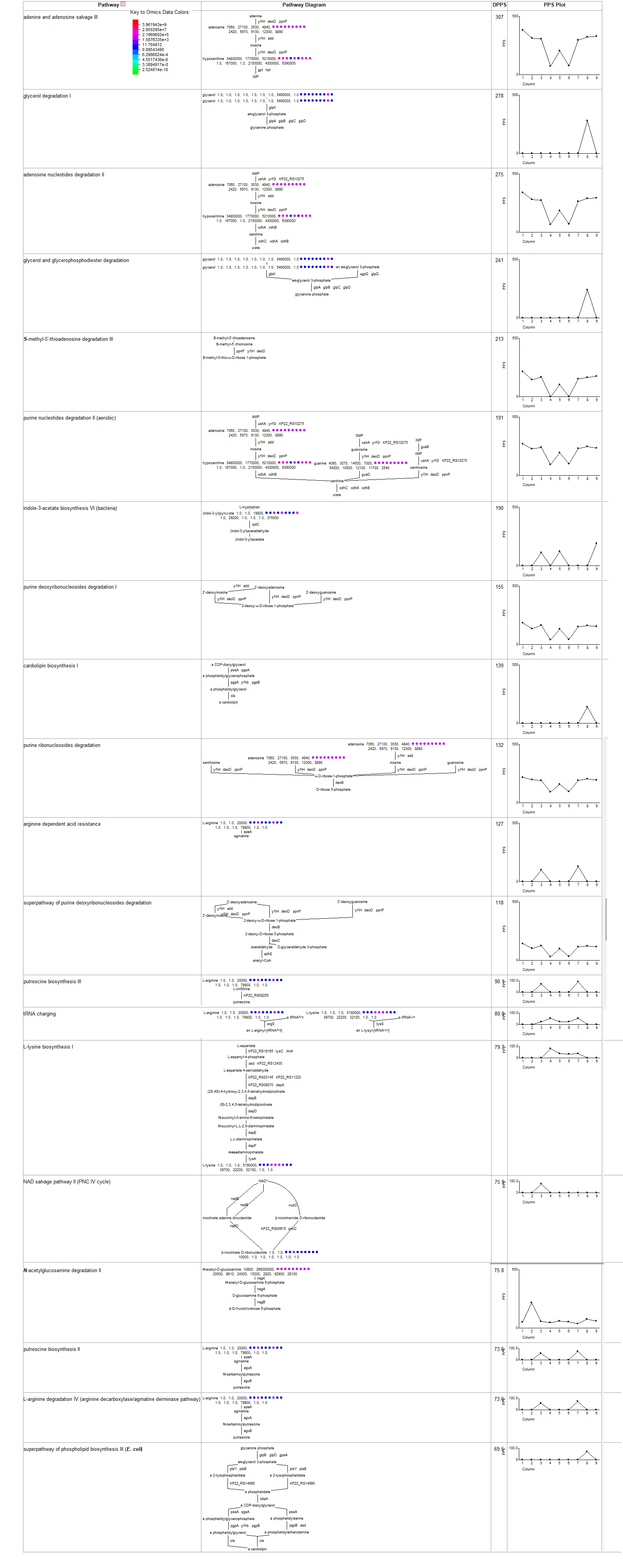


**Supplementary Figure 4.** The pathways detected in *P. betavasculorum* NCPPB 2795 genome database after analysis against compounds present in bacterial medium supplemented with sucrose. The analysis was performed in the PathwayTools software.

**
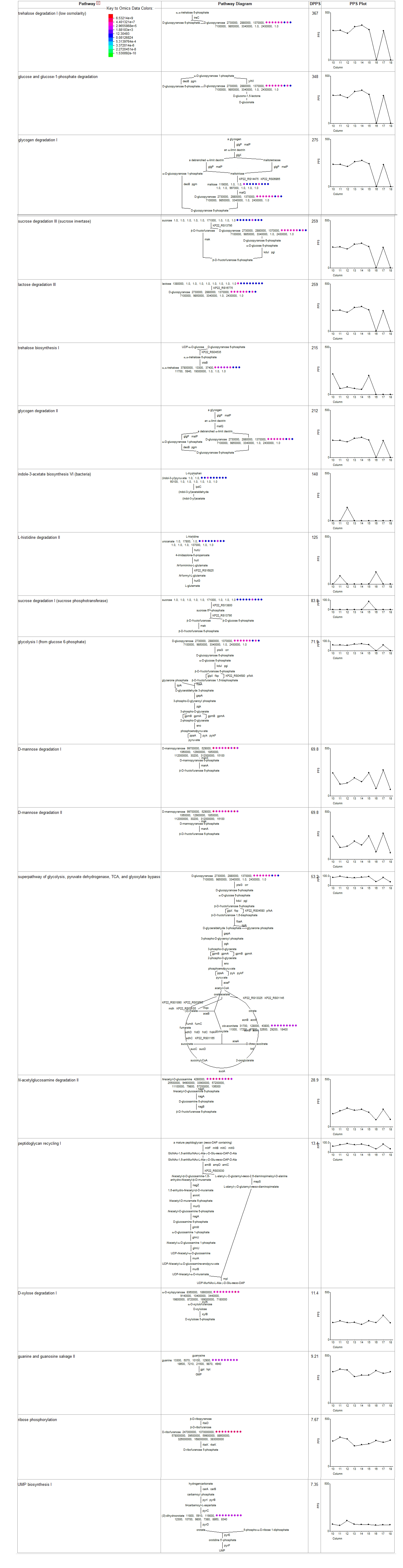
**

**Supplementary Figure 5.** The pathways detected in *P. betavasculorum* NCPPB 2795 genome database after analysis against compounds present in bacterial medium supplemented with xylose. Analysis was performed in the PathwayTools software.


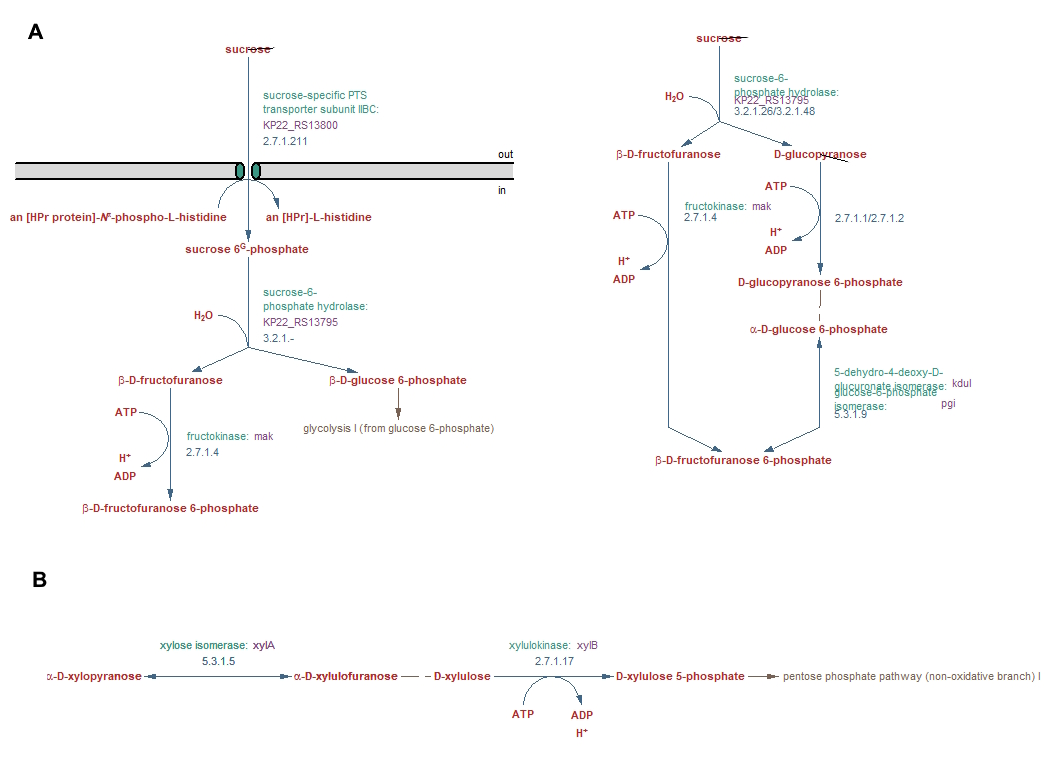


**Supplementary Figure 6.** The pathways of sucrose (A) and xylose (B) degradation in *P. betavasculorum* NCPPB 2795 genome.

**
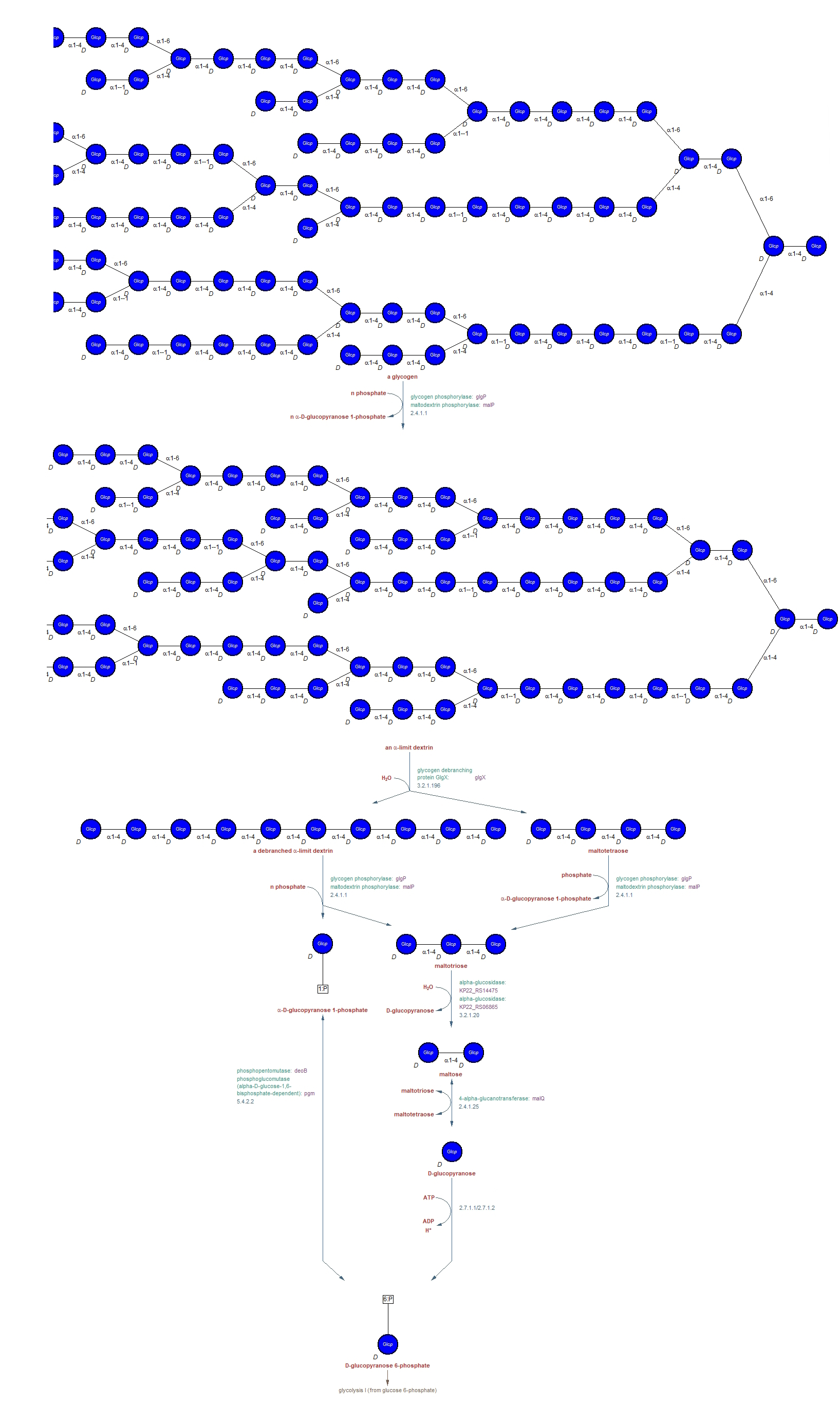
**

**Supplementary Figure 7.** The pathways of glycogen degradation in *P. betavasculorum* NCPPB 2795 genome.

# Multivariate statistics of metabolomic datasets.

PCA models


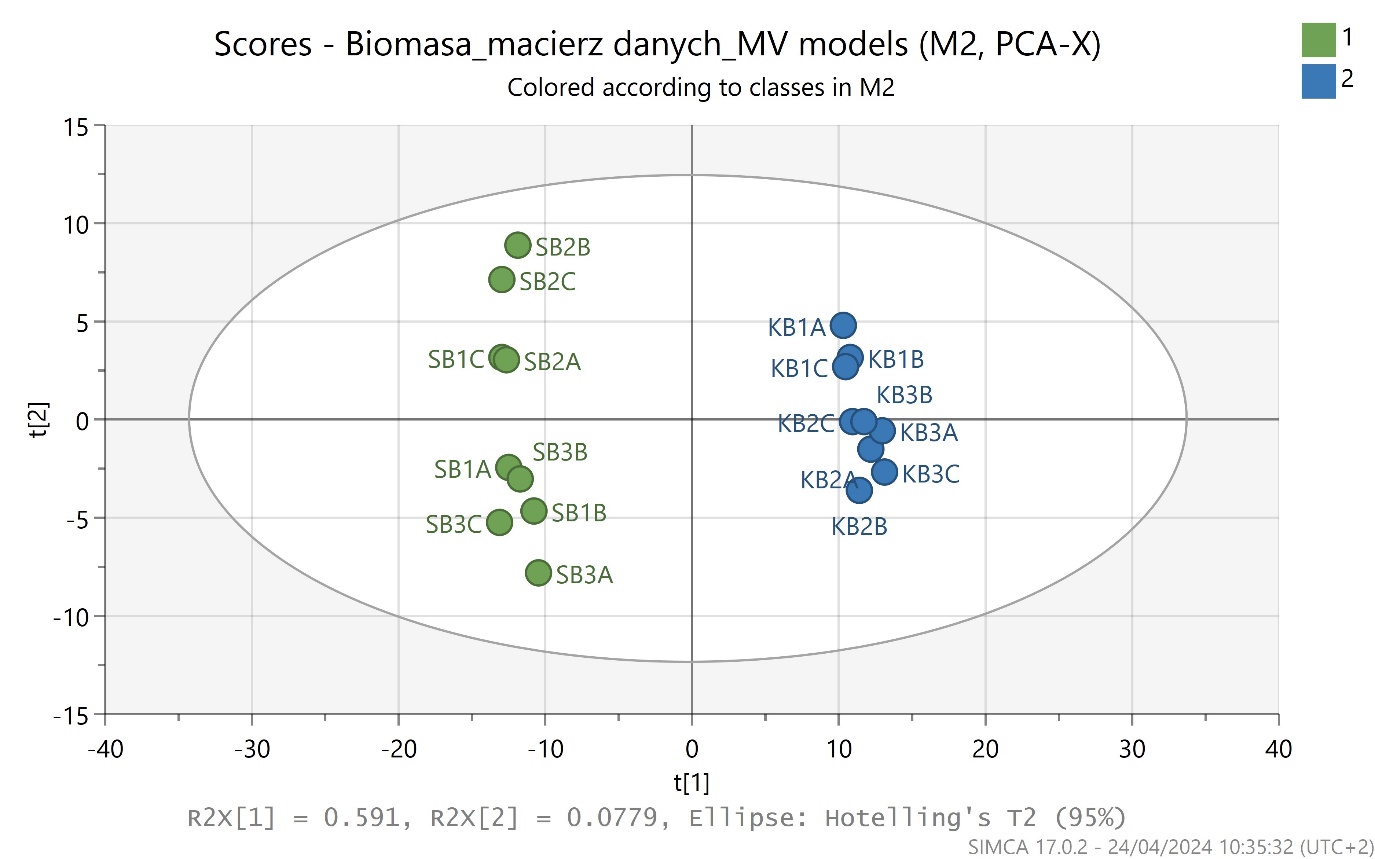


PCA model for dataset of biomass extracts. Green and blue circles correspond to the growth condition in the presence of sucrose and xylose, respectively. R^2^=0.69, Q^2^=0.53.

^
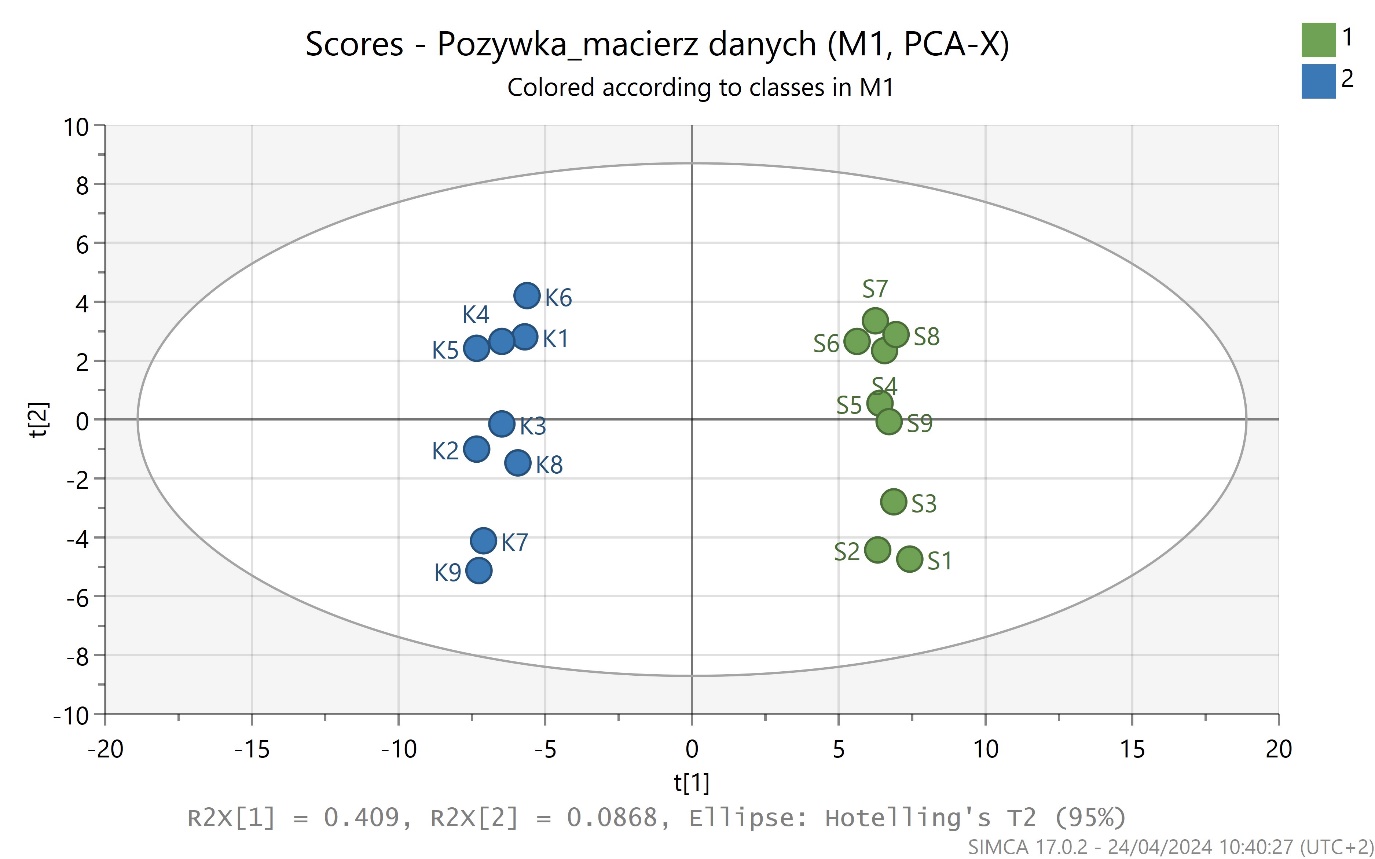
^

PCA model for dataset of media extracts. Green and blue circles correspond to the growth condition in the presence of sucrose and xylose, respectively. R^2^=0.49, Q^2^=0.31.

OPLS-DA models


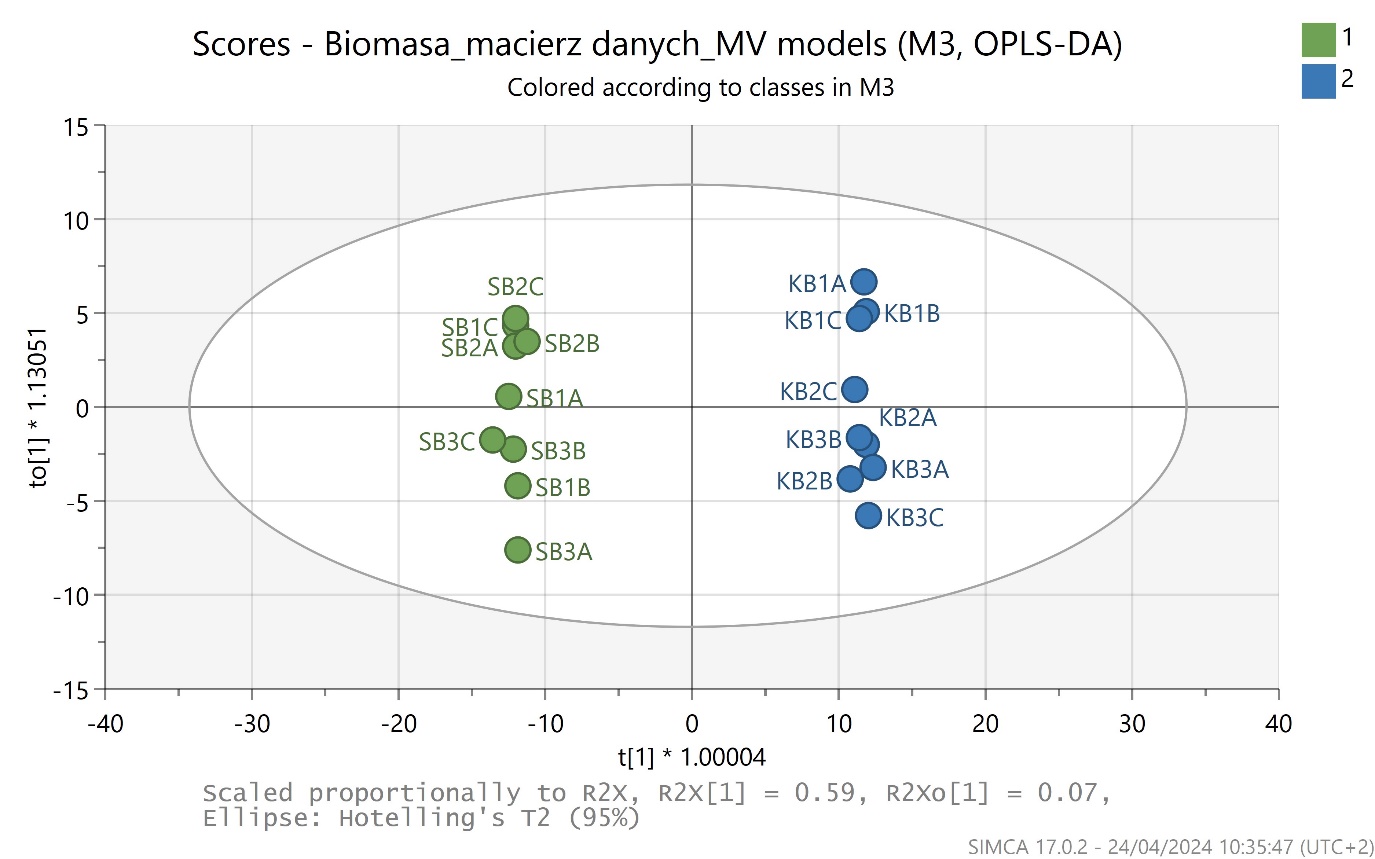


OPLS-DA model for dataset of biomass extracts. Green and blue circles correspond to the growth condition in the presence of sucrose and xylose, respectively. R^2^=0.98, Q^2^=0.93 and
*p* CV-ANOVA=6.4x10^-14^


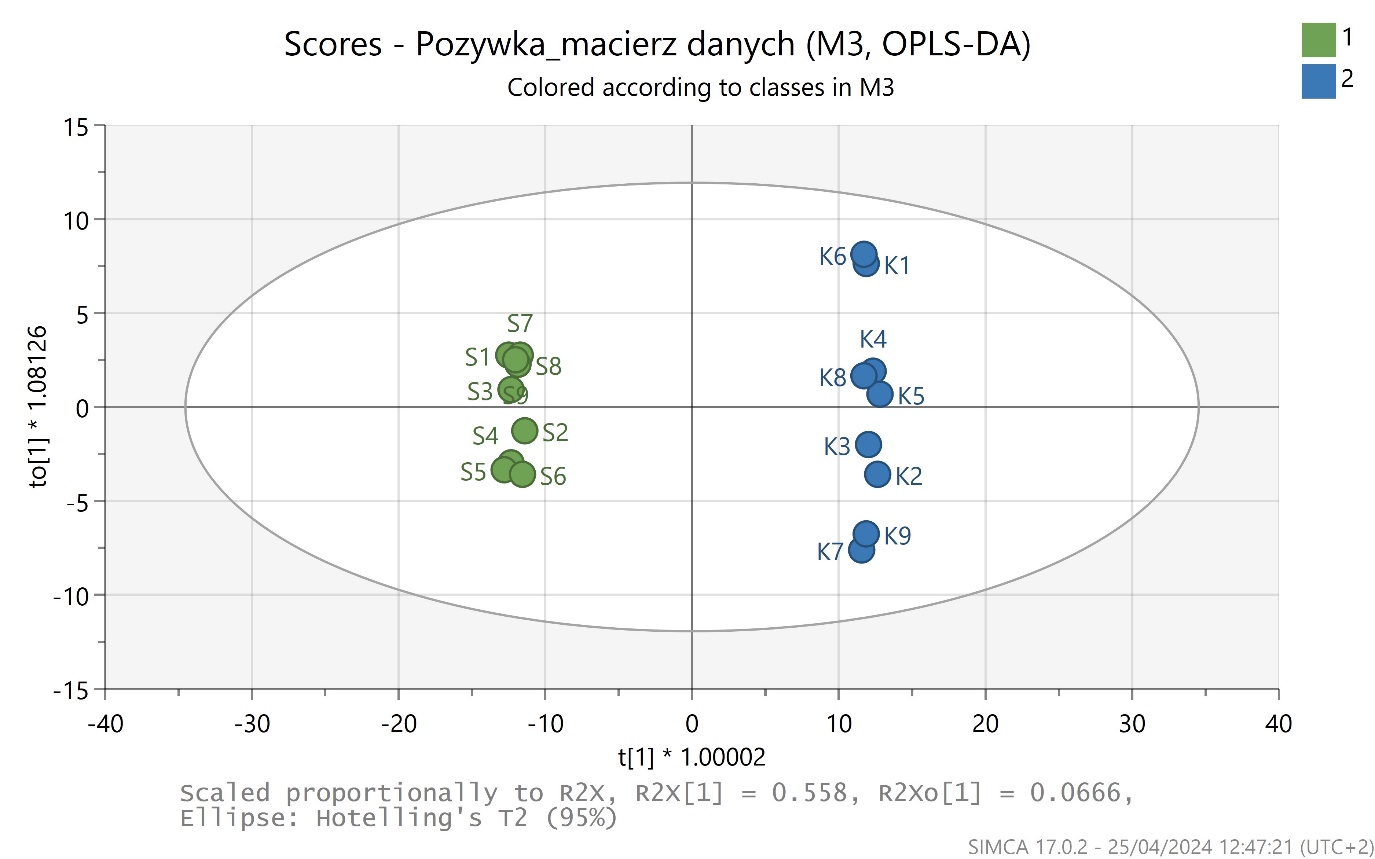


OPLS-DA model for dataset of media extracts. Green and blue circles correspond to the growth condition in the presence of sucrose and xylose, respectively. R^2^=0.99, Q^2^=0.98 and
*p* CV-ANOVA=1.2x10^-14^
